# Dasatinib‐induced hypoglycemia in a patient with acute lymphoblastic leukemia

**DOI:** 10.1002/ccr3.2901

**Published:** 2020-04-27

**Authors:** Michelle D. Lundholm, Gerald A. Charnogursky

**Affiliations:** ^1^ Department of Internal Medicine Loyola University Medical Center Maywood IL USA; ^2^ Department of Medicine Division of Endocrinology Loyola University Health Care System Maywood IL USA

**Keywords:** acute lymphoblastic leukemia, adverse effect, diabetes, hypoglycemia, tyrosine kinase inhibitor

## Abstract

Tyrosine kinase inhibitors can cause significant hypoglycemia in patients with diabetes on other antihyperglycemic medications. These patients should be monitored, and their medications adjusted accordingly.

## Graphical abstract

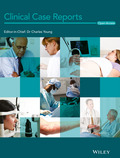

## 1 INTRODUCTION

Tyrosine kinase inhibitors (TKIs) are targeted therapies for use in multiple cancers, including chronic myeloid leukemia (CML), Philadelphia chromosome–positive acute lymphoblastic leukemia (Ph+ ALL), gastrointestinal stromal tumors, and renal cell carcinoma. Prior case reports and studies have suggested that TKIs have metabolic effects. We report a case of profound hypoglycemia in a patient with type 2 diabetes and Ph+ ALL on dasatinib, a second‐generation TKI.

## 2 CASE PRESENTATION

A 65‐year‐old man with hypertension, coronary artery disease, and type 2 diabetes with HbA1c of 10.2% (88 mmol/mol) presented for one month of progressive weakness and dyspnea on exertion. He denied any fevers, chills, night sweats, or chest pain. He had leukocytosis to 203 x 10^9^/L with lymphocyte predominance. A bone marrow biopsy and peripheral flow cytometry were consistent with Ph+ B‐cell ALL and negative for Janus kinase 2 mutation. He was treated with prednisone 120 mg daily. Prior to this hospitalization, he was on a Medtronic Paradigm insulin pump which delivered approximately 120 units of lispro daily. Shortly after starting steroids, his pump malfunctioned so he was transitioned to basal‐bolus insulin and was euglycemic on a regimen of glargine 58 units twice daily and lispro 22 units three times daily (182 units/day of insulin). One week after discharge, his fatigue had mildly improved and he was started on dasatinib 140 mg daily. His other home medications included aspirin 81 mg daily, atorvastatin 40mg daily, losartan 25mg daily, metoprolol 25mg daily, and metformin 1000 mg twice daily.

After 9 days of dasatinib therapy, he began having multiple episodes of hypoglycemia with fingerstick glucoses to 30 mg/dL with weakness and diaphoresis which improved with juice. At this time, his steroid taper had barely decreased from prednisone 120 mg to 90 mg daily. He presented to the emergency department with weakness and shortness of breath, and his serum glucose confirmed hypoglycemia to 36 mg/dL. Laboratory studies were also significant for a hemoglobin of 5.6 g/dL, leukocytes of 3.0 x 10^9^/L, platelets of 29 x 10^9^/L, and a new troponin elevation to 1.66 ng/mL (<0.04 ng/mL). His EKG was unchanged from prior. He was readmitted and transfused blood, and his troponin trended to 1.73 ng/mL. After 11 days of therapy, dasatinib was discontinued for adverse complications including hypoglycemia, pancytopenia, and non‐ST elevation myocardial infarction. He continued to have intermittent episodes of hypoglycemia until his insulin therapy was down‐titrated to a regimen of glargine 10 units daily and lispro 10 units three times daily (40 units/day of insulin). He was discharged home on imatinib 400 mg daily. One week later, he was hyperglycemic with fasting blood sugar to 429 mg/dL despite taking his medications, and his insulin was increased to glargine 35 units daily and lispro 30 units three times daily (125 units/day of insulin). He remained normoglycemic on regimens ranging 120‐190 units/day of insulin thereafter. Within 3 months, his ALL relapsed and he was found to have the T315i mutation, conferring highly aggressive disease with resistance to imatinib. He died in the following weeks from complications of worsening leukemia.

## 3 DISCUSSION

There is evidence from case series and retrospective studies that TKIs can lower blood sugars in the diabetic population. According to one study, 47% of diabetic patients on a TKI were able to discontinue one or more of their antihyperglycemic medications.^1^ TKIs have been shown to improve pancreatic β‐cell survival and increase insulin sensitivity via several mechanisms. Inhibition of c‐Abl prevents upregulation of proapoptotic effectors such as protein kinases, tumor suppressor p73, and caspase‐9, and activation of nuclear factor‐ĸβ decreases islet cell sensitivity to cytokines to reduce inflammation.^2^, ^3^, ^4^ Inhibition of platelet‐derived growth factor receptor decreases the islet cell inflammatory response and increases adipogenesis and adiponectin secretion to enhance insulin sensitivity.^5^ Even in nondiabetic patients there appears to be a modest antihyperglycemic effect, proposed to come from increased insulin sensitivity and reduced hepatic glucose production.^6^

This case presents a patient with type 2 diabetes who developed hypoglycemic episodes requiring modification of his insulin dosing shortly after initiating dasatinib, a second‐generation TKI. This effect ended after discontinuing dasatinib despite starting a first‐generation TKI, imatinib. This highlights how TKIs affect blood glucoses to differing degrees. A study found that dasatinib had the greatest metabolic effect by decreasing random serum blood glucose by 53 mg/dL, averaged between a cohort of diabetic and nondiabetic patients.^1^ In comparison, sunitinib decreased blood sugars by 14 mg/dL, sorafenib by 12 mg/dL, and imatinib by 9 mg/dL on average. TKIs are also shown to disrupt lipid metabolism, and one study demonstrated that dasatinib led to greater increases in triglycerides, LDL, and total cholesterol levels when compared to imatinib.^7^ Dasatinib is unique in that it targets c‐Src, which leads to increased cytosolic free calcium and insulin secretion, as well as decreased reactive oxygen species and reduced islet cell damage.^8^ Across all TKIs, the effect was reversed after discontinuation.

The type of malignancy and its response to TKI treatment also appears to affect the degree of metabolic effect. This case is the first reported in a patient with ALL, and the majority of reports to date have occurred in patients with CML.^9^, ^10^, ^11^ Philadelphia chromosome–positive ALL and CML share the BCR/ABL translocation that makes them particularly susceptible to TKI agents. However, each cancer may be more responsive to some TKIs than others, and it has been noted that the metabolic side effects were greatest in patients who were responsive to treatment.^9^, ^12^ In our patient's case, the reversal of metabolic effect after his switch to imatinib may also serve as an early prognostic indicator that his malignancy would not respond as well to imatinib treatment. This was later confirmed when he relapsed and the T315i mutation was identified. This suggests development of TKI on‐target side effects may be used as clinical indicators of drug efficacy, as they indicate intentional inhibition of receptors associated with oncogenesis.^13^

## 4 CONCLUSIONS

Tyrosine kinase inhibitors can contribute to profound hypoglycemia requiring modification of insulin dosing. Dasatinib has the greatest metabolic effect of the TKIs, especially in patients whose malignancies are responsive to treatment. Further research is needed to understand how these effects differ between TKI drugs and in different forms of cancer. It is critical for prescribers to be aware of the potentially dangerous metabolic side effect from TKIs in their diabetic patients and to monitor glucose levels and adjust antihyperglycemic regimens accordingly.

## CONFLICT OF INTEREST

None declared.

## AUTHOR CONTRIBUTIONS

ML: wrote the manuscript and performed literature research. GC: reviewed and edited the manuscript and is the article guarantor.

## STATEMENT OF ETHICS

The patient has provided informed consent to publish their case.
